# Metabolism and Psychosexual Health in Patients With Polycystic Ovary Syndrome: A Cross-Sectional Study

**DOI:** 10.7759/cureus.88716

**Published:** 2025-07-25

**Authors:** Konstantin Hofmann, Susanne Singer, Susanne Theis, Annette Hasenburg, Roxana Schwab, Christine Skala

**Affiliations:** 1 Department of Obstetrics and Gynecology, University Medical Center of Johannes Gutenberg University Mainz, Mainz, DEU; 2 Department of Quality of Life in Oncology, Comprehensive Cancer Center Mecklenburg-Vorpommern, University Medical Centre Rostock, Rostock, DEU

**Keywords:** anxiety, depression, mental health, metabolism, pcos, psychosexual health

## Abstract

Psychosexual impairments in women with polycystic ovary syndrome (PCOS), often linked to depression, low self-esteem, and negative body image, significantly reduce quality of life. Although metabolic dysfunctions such as insulin resistance and obesity are key features of PCOS, their impact on psychosexual health remains poorly understood. This cross-sectional study aimed to examine the association between metabolic markers and psychosexual functioning in PCOS patients. Participants were recruited from the University Medical Center Mainz and diagnosed using the Rotterdam criteria. Assessments included hormonal and metabolic parameters - such as insulin resistance, body mass index (BMI), fatty liver index (FLI), and visceral adiposity index (VAI) - as well as psychosexual health, using the Female Sexual Function Index (FSFI) and the Hospital Anxiety and Depression Scale (HADS). Logistic regression analyses revealed that visceral adiposity was associated with reduced sexual desire (odds ratio (OR) 2.56, p=0.016), and higher BMI (≥25 kg/m²) with reduced sexual satisfaction (OR 3.56, p=0.049). Elevated insulin resistance (homeostatic model assessment for insulin resistance (HOMA-IR) >5) and FLI (≥60) were associated with increased anxiety and depression, respectively. Overall, higher metabolic burden correlated with impaired sexual function and mental health. These findings highlight the importance of integrating metabolic and psychosexual assessments in PCOS care to better address patients' comprehensive well-being.

## Introduction

The polycystic ovary syndrome (PCOS) is a complex endocrine-metabolic disorder that affects approximately 5%-15% of women worldwide between the ages of 15 and 44 [1]. Thus, it is the most common endocrine condition in premenopausal women globally [2].

The diagnostic standards for PCOS are established in the collaborative PCOS guideline of the European Society of Human Reproduction and Embryology (ESHRE), which draws from the Rotterdam criteria established in 2003. These criteria encompass three components: polycystic ovary morphology or elevated anti-Müllerian hormone (AMH), hyperandrogenemia or clinical androgenization, and irregular cycles. Two of these three criteria must be met to make the diagnosis of PCOS [3]. Nevertheless, PCOS is a complex condition influenced by multiple factors and may manifest with symptom clusters such as obesity, insulin resistance, cardiovascular issues, depression, anxiety, and a range of other disorders [4].

Since the phenotype, severity, and treatment of the condition can vary greatly, there are ongoing discussions in the medical community as to whether all patients diagnosed with PCOS, according to the ESHRE criteria, suffer from the same condition [5]. Even though there are frequent proposals for changes to the diagnostic criteria for PCOS, the ESHRE criteria continue to apply [3].

The pathomechanism of PCOS is the subject of ongoing studies and is not yet fully understood [6]. However, it has been shown that alterations in metabolism constitute a significant contributor to the development of PCOS. Insulin resistance and obesity are prevalent among individuals with PCOS, playing a crucial role in both the severity of symptoms and the prognosis of the disorder [7]. Nevertheless, there are also individuals with PCOS who have a normal weight, both with and without insulin resistance [7].

Psychosexual dysfunctions include issues or challenges that may originate cognitively from depression, low self-esteem, or negative body perception, exerting a noteworthy influence on the affected patients' quality of life and social interactions [3]. Psychosexual health (PSH) is more than the absence of a disease or a sexual disorder. It also includes the ability to fall in love, be aware of one's sexuality, and initiate or maintain a romantic or sexual relationship [8].

Previous findings indicate a negative impact of PCOS on the PSH of patients [9]. In the latest meta-analysis by Pastoor et al., contrary to a previously assumed negative association between obesity and PSH, it was shown that lean PCOS patients have a poorer PSH [9]. However, the specific pathways by which metabolic dysfunction affects psychosexual health in PCOS remain poorly understood. This study aimed to investigate whether a higher metabolic burden - reflected by insulin resistance, body mass index (BMI), and visceral fat - is associated with impairments in psychosexual functioning, including reduced sexual satisfaction and increased anxiety and depression, in women with PCOS.

## Materials and methods

Inclusion and exclusion criteria

At enrollment, the participants were individuals receiving care at the Fertility Center of the Clinic and Polyclinic for Obstetrics and Women's Health at the University Medical Center Mainz. A public announcement of the study was distributed through gynecologists in Mainz and at the PCOS support group in Germany. Gynecologists from private practices then sent patients suspected of having PCOS to our outpatient clinic for follow-up care and inclusion in the study. In addition, patients who had PCOS or were suspected of having PCOS had the opportunity to present themselves at our clinic on their own initiative. The inclusion period took place from spring to winter 2021.

During the study enrollment phase, participants were prohibited from undergoing any PCOS-related therapy. If individuals were already undergoing such therapy, it was temporarily halted before inclusion. Additionally, a present intent to conceive resulted in non-inclusion in the study. However, a general desire for children in the future did not lead to exclusion. To minimise confounders that could impact psychosexual health outcomes, individuals with a history of mental health disorders or severe comorbidities that might interfere with assessment were also excluded. A detailed overview of the inclusion and exclusion criteria can be found in Table 1.

**Table 1:** Criteria for Inclusion and Exclusion of the Study Participants PCOS: Polycystic ovary syndrome; ESHRE: European Society of Human Reproduction and Embryology

Category	Criterion
Inclusion criteria	Age ≥18 years
	Diagnosis of PCOS confirmed using ESHRE criteria after exclusion of differential diagnoses
	No current PCOS-related therapy at the time of inclusion (if present, therapy was paused)
Exclusion Criteria	Age <18 years
	Ongoing PCOS-related therapy that could not be paused
	Present intent to conceive
	Mental health disorders in the patient’s history
	Severe comorbidities that could interfere with the assessment
	Differential diagnoses such as adrenogenital syndrome, hypercortisolism, or hormone-secreting tumors

Parameters

Each participant underwent an evaluation of hormone status (anti-Müllerian hormone (electrochemiluminescence immunoassay (ECLIA)), androstenedione (liquid chromatography with tandem mass spectrometry (LC-MS-MS)), dehydroepiandrosterone sulfate (ECLIA), estradiol (ECLIA), follicle-stimulating hormone [chemiluminescence microparticle immunoassay (CLIA)), luteinizing hormone (CLIA), progesterone (CLIA), prolactin (CLIA), sex hormone-binding globulin (CLIA), testosterone (CLIA), thyroid-stimulating hormone (CLIA), 17-OH progesterone (LC-MS-MS). If hormonal therapy had previously been used, this was interrupted for a period of three months before the analysis. All hormone analyses were carried out in the follicular phase on cycle days 1 to 5. To evaluate hirsutism, patients completed the Ferriman-Gallwey questionnaire. A score exceeding 7 was deemed indicative of pathological hirsutism [10].

Metabolic assessment included examinations of liver and kidney values, serum lipids, C-reactive protein, and a fasting 75 g oral glucose tolerance test (oGTT/insulin). Waist circumference (WC) was measured between the last rib and the level of the iliac crest.

The World Health Organization defines body mass index (BMI) ≤24.9 kg/m^2^, 25-29.9 kg/m^2^, ≥30 kg/m^2^ and ≥35 kg/m^2^ as healthy weight, overweight and obesity I and II.

Homeostatic model assessment for insulin resistance (HOMA-IR)

Homeostatic model assessment for insulin resistance (HOMA-IR) was calculated according to the formula:

$\text{HOMA-IR} = \frac{\text{Fasting Insulin (mU/L)} \times \text{Fasting Glucose (nmol/L)}}{22.5}$

An index surpassing 2 was considered indicative of potential insulin resistance, while a range of 2.5 to 5 was classified as probable insulin resistance, and a value exceeding 5 was categorized as severe insulin resistance [11].

Fatty liver index

The fatty liver index (FLI) indicates the likelihood of an individual having hepatic steatosis. Bodogni et al. established an algorithm comprising four parameters (BMI, WC, triglycerides (TG), and gamma-glutamyltransferase (GGT)) selected from various variables to assess this likelihood [12].

FLI is calculated as:

 $\text{FLI} = \frac{e^x}{1 + e^x} \times 100$

$\text{where } x = 0.953 \times \log_e(\text{TG}) + 0.139 \times \text{BMI} + 0.718 \times \log_e(\text{GGT}) + 0.053 \times \text{WC} - 15.745$

with TG measured in mmol/l, BMI in kg/m^2^, GGT in U/L, and WC in centimeters.

The values of FLI vary from 0 to 100. FLI  ≥ 30 indicates a low likelihood of fatty liver, and FLI ≥ 60 indicates a high degree of probability of the presence of fatty liver [12].

Visceral adiposity index

While the BMI is widely utilized in clinical settings to evaluate a patient's nutritional status, it lacks information on the distribution of fat and muscle tissue. The visceral adiposity index (VAI) serves as a reliable and simple surrogate marker [13]. To date, a definitive cut-off for a pathological VAI has not been defined. Studies on VAI use the marker to predict Type 2 diabetes mellitus (T2DM) rather than insulin resistance, which plays an important role, especially in PCOS patients [13,14].

The calculation of the VAI involves using BMI, WC, and certain biochemical parameters, namely TG and HDL cholesterol [15]. The version of the formula for women was used to calculate the VAI [15]:

$\text{VAI} = \left( \frac{\text{WC (cm)}}{36.58 + (1.89 \times \text{BMI})} \right) \times \left( \frac{\text{TG}}{0.81} \right) \times \left( \frac{1.52}{\text{HDL}} \right)$

Assessment of female sexual function

The Female Sexual Function Index (FSFI), a self-reporting tool, consists of 19 questions for assessing female sexuality [16]. It can be divided into six subcategories [16]: desire, arousal, lubrication, orgasm, satisfaction, and pain. Each of these domains has its own cut-off score, which can indicate a deviation of sexual function into the pathological range. In addition, a total score cut-off is used to assess the overall sexual function [17]. These subcategories and their respective cut-off values are summarised in Table 2. For this study, the validated German version of the FSFI was used [18].

**Table 2:** Female Sexual Function Index (FSFI) Subcategories and Cut-Off Scores [16,17]

Subcategory	Description	Cut-Off Score
Desire	Wish to engage in a sexual experience, receptivity towards sexual initiation	<4.28
Arousal	Levels and types of arousal, signs of attention or excitement	<5.08
Satisfaction	Level of contentment with actual sexual life	<5.04
Lubrication	Presence, quantity, or absence of vaginal lubrication during sexual excitement	<5.45
Orgasm	Ability to attain orgasms	<5.05
Pain	Sensation of discomfort during intercourse	<5.51
Total	The aggregate value from the subcategories	<26.55

Mental health

Anxiety and depression were assessed with the Hospital Anxiety and Depression Scale (HADS) [19]. The HADS is a self-report instrument that comprises two subscales for anxiety and depression, each consisting of seven questions. Respondents provided one of four possible answers for each item, ranging from 0 (absence of symptoms) to 3 (maximum symptoms). Elevated scores indicate higher levels of anxiety or depression. The threshold for both symptom categories is a score of 8 or more. Scores in the range of 8-10 indicate mild anxiety or depression, 11-14 suggest moderate anxiety or depression, and 15-21 indicate severe anxiety or depression [20]. In this study, we utilized its validated German version [19]. 

Statistical analysis and ethical considerations

The data underwent descriptive and exploratory data analysis, employing IBM SPSS Statistics Version 29 (IBM Corp, Armonk, NY). Participant characteristics were described using mean±standard deviation and percentage. Univariate logistic regression analyses were utilized to evaluate the association of markers related to PCOS, with a specific focus on metabolic markers, on PSH. Variables with p-values less than 0.05 in the univariate regression model were then entered into the final model multivariate logistic regression by backward stepwise selection to determine the independence of the variables mentioned above for predicting an impairment of PSH. Prior to their inclusion in the multivariate model, the parameters to be included were checked for correlation. If the Pearson correlation coefficient or Spearman correlation coefficient was greater than 0.7, these parameters were not simultaneously included in the model.

The selection of confounders was performed from the following clinically plausible factors: age, Ferriman-Gallwey Score, and partnership. The model was adjusted using a change-in-estimate approach. Potential confounders were included in the final logistic regression model if they changed the odds ratio (OR) by more than 10% [21]. The OR, p-value, and 95% confidence interval (95% CI) were utilized to estimate effects.

The Ethics Committee of the Rhineland-Palatinate Medical Association (Landesärztekammer Rheinland-Pfalz) approved the study (2021-15680_1), which was conducted according to the requirements of the Declaration of Helsinki. Informed consent was obtained from all participants prior to their inclusion in the study.

## Results

Anthropometric and serological characteristics

The study enrolled 77 patients with PCOS, with an average age of 26.9 years and a BMI of 28.0 kg/m². A total of 32.5% of the participants in the study exhibited a HOMA-IR greater than 2.5, suggesting a potentially pathological condition. Moreover, 36.1% displayed an FLI equal to or greater than 30, categorizing them within the pathological range for hepatic steatosis. Table 3 provides an overview of the baseline demographics of the study sample. Additional data can be referenced in the appendices.

**Table 3:** Anthropometric and serological characteristics of the study population BMI: Body Mass Index; HDL: high-density lipoprotein cholesterol; HOMA-IR: Homeostatic Model Assessment for Insulin Resistance; LDL: low-density lipoprotein cholesterol; PCOS: polycystic ovary syndrome; SD: standard deviation

Characteristics	Overall
N	77
Age (years) Mean ± SD	26.9±4.4
Weight (kg), Mean±SD	78.2±21.1
BMI (kg/m²), Mean±SD	28.0±7.0
Healthy weight BMI (18.5-24.9 kg/m²)	36 (46.8%)
Overweight BMI (25.0-29.9 kg/m²)	17 (22.1%)
Obesity I BMI (30.0-34.9 kg/m²)	7 (9.1%)
Obesity II BMI (≥35.0 kg/m²)	17 (22.1%)
PCOS Phenotype	
A (hyperandrogenism + ovulatory dysfunction + PCOM)	52 (67.5%)
B (hyperandrogenism + ovulatory dysfunction)	11 (14.3%)
C (hyperandrogenism + PCOM)	11 (14.3%)
D (ovulatory dysfunction + PCOM)	3 (3.9%)
Waist circumference (cm)	83.76±16.49
HOMA-IR, Mean±SD	2.21±1.87
< 2 (normal)	44 (57.1%)
2-2.5 (possible insulin resistance)	8 (10.4%)
2.5-5 (likely insulin resistance)	18 (23.4%)
> 5 (severe insulin resistance)	7 (9.1%)
Ferriman-Gallwey Score, Mean ± SD	14.3 ± 5.9
<7 normal	6 (7.8%)
≥7 pathological hirsutism	71 (92.2%)
HDL (mg/dl) Mean ± SD > 40.0 (normal)	58.6±12.4
LDL (mg/dl) Mean ± SD < 160.0 (normal)	103.1±33.7
Triglyceride (mg/dl) Mean ± SD < 150.0 (normal)	109.2±92.7
Cholesterol (mg/dl) Mean ± SD < 200.0 (normal)	184.2±41.9
Free Androgen Index (FAI) Mean ± SD	4.3±3.0
<5.5 normal	23 (26 %)
≥5.5 elevated	54 (74.0%)
Visceral Adiposity Index (VAI) Mean ± SD	3.3±2.6
Fatty Liver Index (FLI) Mean ± SD	32.8±34.6
<30 no hepatic steatosis	31 (36.1%)
≥30 low likelihood of hepatic steatosis	26 (36.1%)
≥60 probable hepatic steatosis	20 (27.8%)
Partnership	
Yes	41 (53.2 %)
No	36 (46.8%)

Sexual function

**Figure 1:**
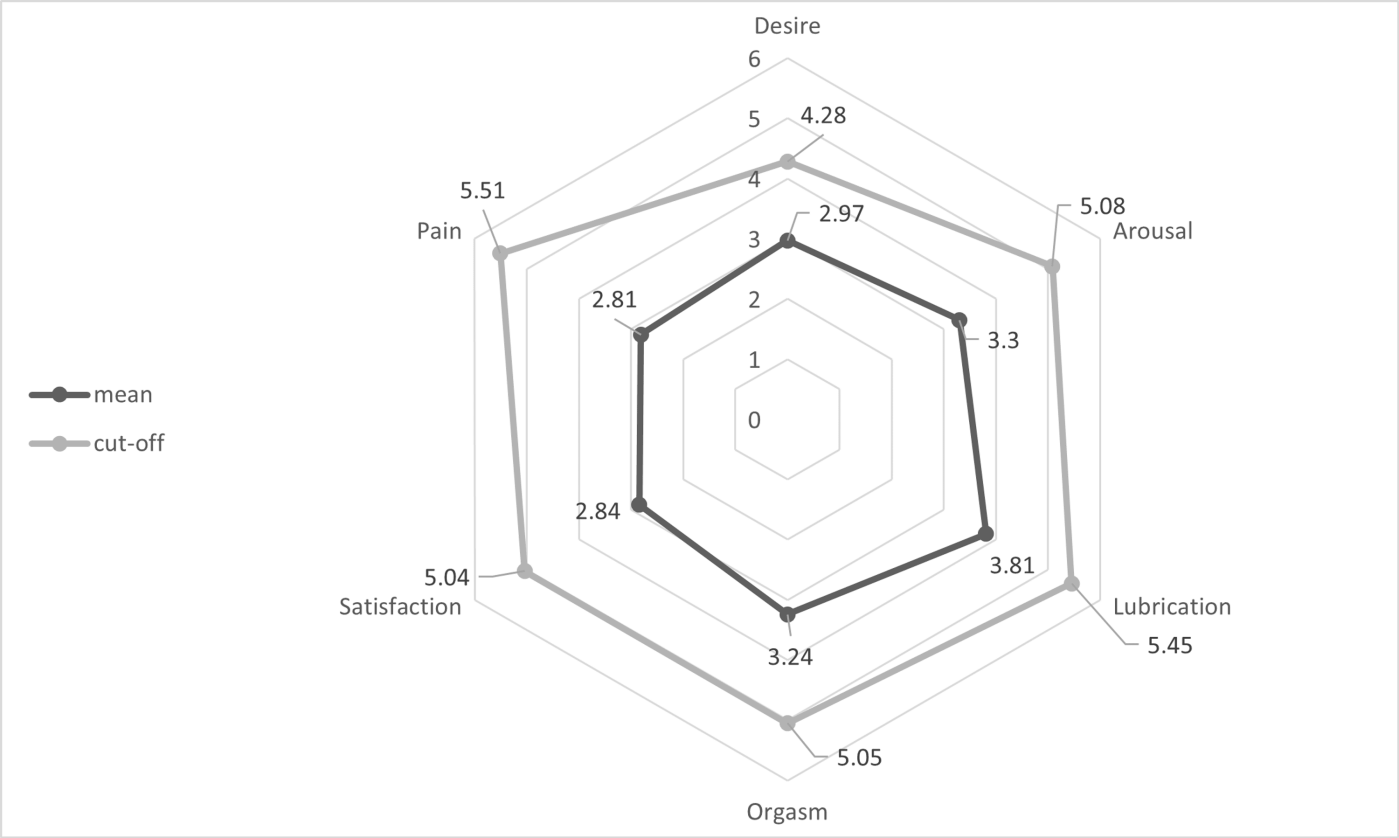
Results of the FSFI subcategories

The cut-off values in the subcategories of the FSFI indicate an impairment of sexual function. In the overall study population, consistently reduced values were observed in all subcategories: 81.8% (63) of the patients were in the pathological range of FSFI-Desire, 79.2% (61) of FSFI-Arousal, 67.5% (52) of FSFI-Lubrication, 68.8% (53) of FSFI-Orgasm, 81.8% (63) of FSFI-Satisfaction, 75.3% (58) of FSFI-Pain, and 71.4% (55) of FSFI-Total (Figure 1)

Mental health

The study participants recorded an average anxiety score of 9.2. Notably, 28.6% showed mild symptoms of anxiety, 15.6% moderate symptoms, and 16.9% severe symptoms of anxiety.

**Figure 2:**
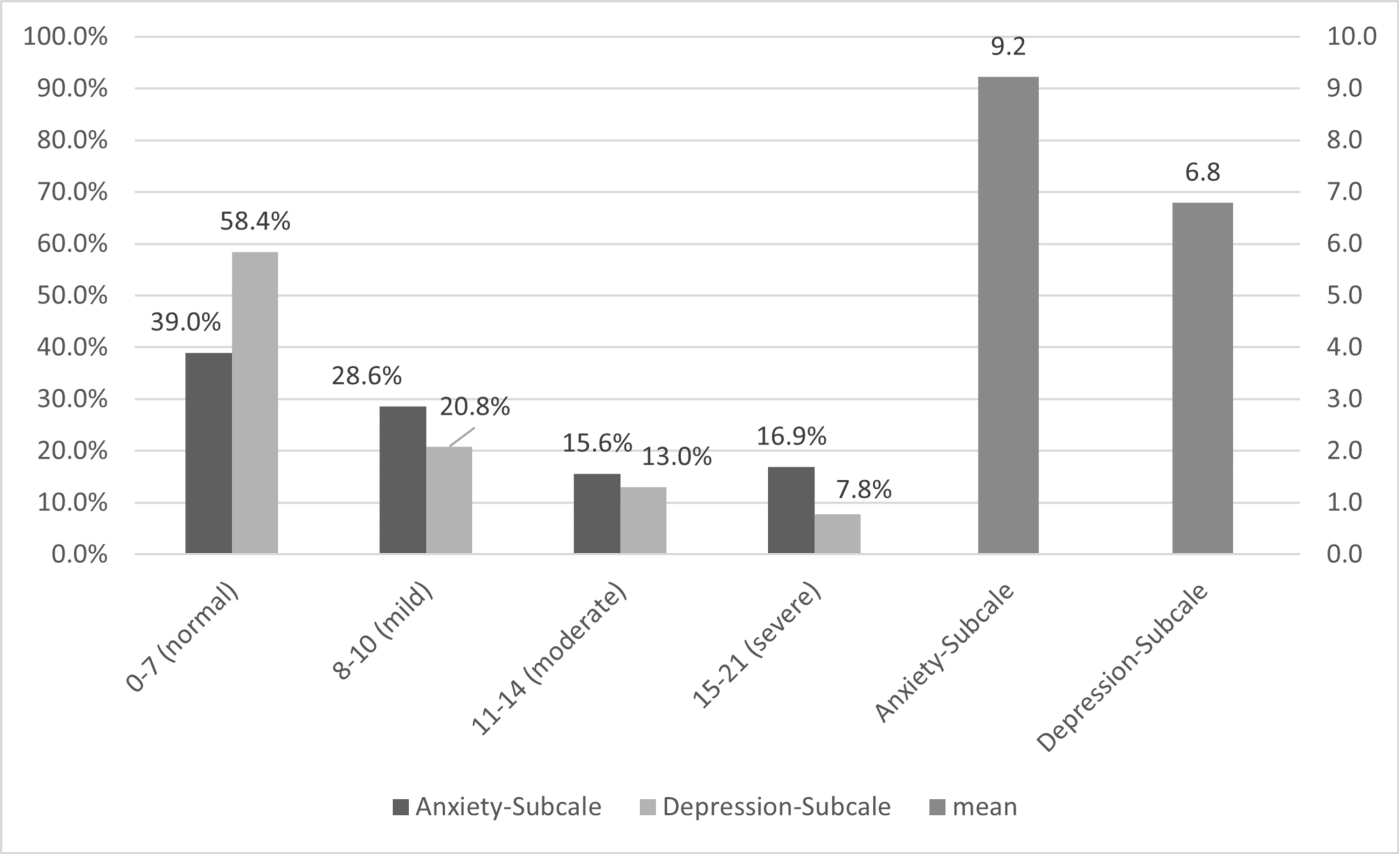
Proportion of patients with impaired mental health using the Hospital Anxiety and Depression Scale (HADS)

The average depression score was 6.8, but 20.8% reported mild, 13.0% moderate, and 7.8% of the participants had severe symptoms of depression. Of the patients, 39.0% and 58.4% showed normal values of HADS-Anxiety and HADS-Depression, respectively (Figure 2).

Univariate and multivariate analysis: sexual function

To investigate the relationship between metabolism and the FSFI, univariate and multivariate analyses using logistic regression were conducted (Table 4).

The analysis revealed a significant association between VAI (OR 2.56; CI: 1.19-5.56; p=0.016), FLI≥30 (OR 8.82; CI: 1.08-72.37; p=0.048), and BMI≥25kg/m² (OR 5.57; CI: 1.41-21.99; p=0.014) with FSFI-Desire (Table 4). The multivariate analysis for FSFI-Desire<4.28 showed a significant association for VAI (OR 2.56; CI: 1.19-5.56; p=0.016). The final regression model (n=71) demonstrated a sensitivity of 81.7% for predicting FSFI-Desire levels<4.28.

For FSFI-Satisfaction, BMI≥25kg/m² (OR 3.56; CI: 1.01-12.59; p=0.049) and no partnership (OR 0.14; CI: 0.03-0.69; p=0.015) were statistically significant (Table 4). The adjustment did not result in a change in the OR of more than 10%, indicating that a multivariate model was not necessary.

**Table 4:** Association between sexual function and metabolism; univariate logistic regression BMI: Body mass index, CI: confidence interval, FLI: fatty liver index, FSFI: Female Sexual Function Index, HOMA-IR: homeostatic model assessment for insulin resistance, OR: odds ratio, VAI: visceral adiposity index

FSFI	Desire	Arousal	Lubrication	Orgasm	Satisfaction	Pain <5.51	Total <26.55
	<4.28	<5.08	<5.45	<5.05	<5.04		
HOMA-IR	OR 0.71 (CI: 0.42-1.19) p=0.19	OR 1.07 (CI: 0.82-1.41) p=0.61	OR 1.00 (CI: 0.78-1.30) p=0.98	OR 0.90 (CI: 0.67-1.21) p=0.49	OR 0.83 (CI: 0.54-1.26) p=0.38	OR 0.90 (CI: 0.65-1.24) p=0.52	OR 0.89 (CI: 0.66-1.22) p=0.47
HOMA-IR >2	OR 3.33 (CI: 0.85-13.11) p=0.085	OR 0.96 (CI: 0.32-2.90) p=0.94	OR 0.73 (CI: 0.28-1.92) p=0.53	OR 1.38 (CI: 0.51-3.70) p=0.52	OR 2.13 (CI: 0.60-7.52) p=0.24	OR 2.61 (CI: 0.83-8.20) p=0.10	OR 1.921(CI: 0.68-5.45) p=0.22
HOMA-IR >2.5	OR 3.45 (CI: 0.71-16.79) p=0.13	OR 0.75 (CI: 0.24-2.38) p=0.63	0.79 (CI: 0.29-2.16) p=0.65	OR 1.68 (CI: 0.57-4.94) p=0.35	OR 1.97 (CI: 0.50-7.80) p=0.34	OR 2.13 (CI: 0.63-7.25) p=0.23:	OR 1.41 (CI: 0.47-4.19) p=0.54
HOMA-IR >5	OR 1.37 (CI: 0.15-12.36) p=0.78	OR 0.63 (CI: 0.11-3.57) p=0.60	OR 1.22 (CI: 0.22-6.79) p=0.82	OR 1.15 (CI: 0.21-6.37) p=0.88	OR 1.37 (CI: 0.15-12.36) p=0.78	OR 0.80 (CI: 0.14-4.52) p=0.80	OR 1.0 (CI: 0.18-5.58) p=1.0
VAI	OR 2.56 (CI: 1.19-5.56) p=0.016	OR 1.02 (CI: 0.82-1.28) p=0.83	OR 1.04 (CI: 0.85-1.27) p=0.72	OR 0.94 (CI: 0.79-1.14) p=0.56	OR 1.09 (CI: 0.83-1.45) p=0.54	OR 1.23 (CI: 0.91-1.67) p=0.18	OR 1.12 (CI: 0.88-1.43) p=0.34
FLI	OR 0.98 (CI: 0.95-1.00) p=0.069	OR 1.01 (CI: 0.99-1.02) p=0.56	OR 1.01 (CI: 0.99-1.02) p=0.33	OR 1.00 (CI: 0.99-1.02) p=0.96	OR 0.99 (CI: 0.97-1.01) p=0.33	OR 0.99 (CI: 0.98-1.01) p=0.37	OR 0.10 (CI: 0.98-1.01) p=0.58
								FLI ≥30	OR 8.82 (CI: 1.08-72.37) p=0.043	OR 0.93 (CI: 0.29-2.93) p=0.90	OR 0.70 (CI: 0.26-1.92) p=0.4	OR 1.20 (CI: 0.43-3.36) p=0.72	OR 2.13 (CI: 0.53-8.57) p=0.29	OR 1.66 (CI: 0.52-5.32) p=0.40	OR 1.61 (CI: 0.54-4.85) p=0.40
FLI ≥60	OR 5.70 (CI: 0.69-47.10) p=0.11	OR 0.81 (CI: 0.24-2.70) p=0.73	OR 0.67 (CI: 0.23-1.95) p=0.46	OR 0.90 (CI: 0.30-2.67) p=0.85	OR 2.42 (CI: 0.49-12.02) p=0.28	OR 1.47 (CI: 0.42-5.17) p=0.55	OR 1.94 (CI: 0.56-6.71) p=0.29
BMI	OR 0.91 (CI: 0.82-1.02) p=0.093	OR 1.02 (CI: 0.94-1.10) p=0.60	OR 1.03 (CI: 0.96-1.10) p=0.42	OR 1.02 (CI: 0.95-1.09) p=0.67	OR 0.96 (CI: 0.87-1.05) p=0.37	OR 0.99 (CI: 0.92-1.07) p=0.82	OR 0.98 (CI: 0.91-1.06) p=0.59
BMI ≥25kg/m²	OR 5.57 (CI: 1.41-21.99) p=0.014	OR 1.62 (CI: 0.53-4.91) p=0.40	OR 1.37 (CI: 0.53-3.56) p=0.52	OR 0.95 (CI: 0.36-2.49) p=0.91	OR 3.56 (CI: 1.01-12.59) p=0.049	OR 2.43 (CI: 0.83-7.07) p=0.10	OR 2.63 (CI: 0.94-7.30) p=0.064
BMI ≥30kg/m²	OR 7.48 (CI: 0.92-60.90) p=0.060	OR 0.10 (CI: 0.30-3.27) p=0.99	OR 0.72 (CI: 0.26-1.96) p=0.53	OR 0.66 (CI: 0.24-1.82) p=0.42	OR 1.83 (CI: 0.46-7.29) p=0.39	OR 0.98 (CI: 0.32-2.98) p=0.97	1.79 (CI: 0.57-5.62) p=0.32
BMI ≥35 kg/m²	OR 4.43 (CI: 0.54-36.56) p=0.17	OR 0.81 (CI: 0.22-2.94) p=0.75	OR 0.45 (CI: 0.15-1.34) p=0.15	OR 0.79 (CI: 0.25-2.45) p=0.68	OR 1.05 (CI: 0.26-4.28) p=0.95	OR 1.70 (CI: 0.43-6.69) p=0.45	OR 0.95 (CI: 0.29-3.10) p=0.93
								Age	OR 0.85 0.72-1.01) p=0.065	OR 0.84 (CI: 0.72-0.10) p=0.040	OR 0.93 (CI: 0.82-1.05) p=0.22	OR 1.02 (CI: 0.92-1.14) p=0.70	OR 0.94 (CI: 0.82-1.00) p=0.43	OR 0.98 (CI: 0.87-1.10) p=0.71	OR 0.95 (CI: 0.84-1.07) p=0.39
								No partnership	OR 1.67 (CI: 0.52-5.37) p=0.39	OR 0.86 (CI: 0.29-2.60) p=0.79	OR 0.52 (CI: 0.20-1.39) p=0.19	OR 0.58 (CI: 0.22-1.55) p=0.28	OR 0.14 (CI: 0.03-0.69) p=0.015	OR 0.22 (CI: 0.064-0.73) p=0.014	OR 0.42 (CI: 0.15-1.19) p=0.10
Ferriman Gallwey-Score >7	OR 2.46 (CI: 0.40-14.98) p=0.33	OR 4.46 (CI: 0.81-24.66) p=0.086	OR 2.23 (CI: 0.42-11.92) p=0.35	OR 5.10 (CI: 0.87-30.07) p=0.072	OR 2.46 (CI: 0.40-14.98) p=0.33	OR 7.47 (CI: 1.25-44.74) p=0.028	OR 5.89 (CI: 0.99-34.91) p=0.051

Regarding FSFI-Pain, whilst no partnership (OR 0.22; CI: 0.064-0.73; p=0.014) and Ferriman-Gallwey >7 (OR 7.47; CI 1.25-44.74; p=0.028) emerged as relevant univariate factors (Table 4). No metabolic marker played a significant role in this category. No metabolic marker showed significance in the univariate analysis regarding FSFI-arousal, FSFI-lubrication, FSFI-orgasm, FSFI-total, or FSFI-total (Table 4).

Univariate and multivariate analysis: mental health

No confounder met the criterion of the change-in-estimate approach. Considering HADS-Anxiety >8, HOMA-IR >5 (OR 11.49; CI: 1.32-100; p=0.028) and Ferriman-Gallwey >7 (OR 9.20; CI: 1.02-83.16; p=0.048), they presented statistically significant results in the univariate regression (Table 5).

HADS-depression>8 showed a statistically significant association with HOMA-IR>2 (OR 2.57; CI: 1.01-6.54; p=0.047), FLI ≥60 (OR 3.51; CI: 1.19-10.35; p=0.023), FSFI-Pain (OR 3.50, CI: 1.04-11.82; p=0.044) and FSFI-Total (OR 3.28; CI: 1.06-10.14; p=0.039) in univariate analysis. In the multivariate model (n=72 cases, sensitivity 56.9%), FLI>60 (OR 3.49; CI: 1.14-10.65; p=0.029) remained statistically significant (Table 5).

**Table 5:** Association between psychosexual health, anxiety, depression and metabolism; univariate logistic regression BMI: Body mass index, CI: confidence interval, FLI: fatty liver index, FSFI: Female Sexual Function Index, HADS: Hospital Anxiety and Depression Scale, HOMA-IR: homeostatic model assessment for insulin resistance, OR: odds ratio, VAI: visceral adiposity index

	Anxiety	Depression
HOMA-IR	OR 1.15 (CI: 0.90-1.48; p=0.27	OR 0.95 (CI: 0.75-1.22) p=0.71
HOMA-IR >2	OR 0.78 (CI: 0.31-1.95) p=0.590	OR 2.57 (CI: 1.01-6.54) p=0.047
HOMA-IR >2.5	OR 0.57 (CI: 0.22-1.51) p=0.26	OR 1.82 (CI: 0.72-4.95) p=0.20
HOMA-IR >5	OR 11.49 (CI: 1.32-100) p=0.028	OR 1.88 (CI: 0.34-10.3) p=0.47
VAI	OR 1.12 (CI: 0.93-1.36) p=0.23	OR 0.94 (CI: 0.79-1.13) p=0.52
FLI	OR 1.00 (CI: 0.99-1.02) p=0.75	OR 0.99 (CI: 0.98-1.00) p=0.13
FLI ≥30	OR 1.03 (CI: 0.38-2.76) p=0.96	OR 1.56 (CI: 0.59-4.11) p=0.37
FLI ≥60	OR 0.94 (CI: 0.33-2.69) p=0.91	OR 3.51 (CI: 1.19-10.35) p=0.023
BMI	OR 1.00 (CI: 0.94-1.07) p=0.96	OR 0.95 (CI: 0.89-1.02) p=0.14
BMI ≥25 kg/m²	OR 0.99 (CI: 0.40-2.49) p=0.99	OR 2.39 (CI: 0.94-6.09) p=0.069
BMI ≥30 kg/m²	OR 1.09 (CI: 0.41-2.95) p=0.86	OR 2.12 (CI: 0.79-5.63) p=0.13
BMI ≥35 kg/m²	OR 0.89 (CI: 0.30-2.66) p=0.83	OR 2.47 (CI: 0.82-7.41) p=0.11
Age	OR 0.96 (CI: 0.87-1.07) p=0.50	OR 1.03 (CI: 0.93-1.15) p=0.55
No partnership	OR 0.80 (CI: 0.32-2.01) p=0.63	OR 0.80 (CI: 0.32-1.99) p=0.63
Ferriman Gallwey>7	OR 9.20 (CI: 1.02-83.16) p=0.048	OR 1.46 (CI: 0.25-8.52) p=0.67
FSFI Desire< 4.28	OR 1.74 (CI: 0.54-5.58) p=0.35	OR 2.00 (CI: 0.57-7.06) p=0.28
FSFI Arousal<5.08	OR 0.93 (CI: 0.30-2.88) p=0.89	OR 1.24 (CI: 0.40-3.84) p=0.71
FSFI Lubrication<5.45	OR 1.07 (CI: 0.40-2.83) p=0.90	OR 1.10 (CI: 0.42-2.91) p=0.85
FSFI Orgasm<5.05	OR 1.51 (CI: 0.57-4.03) p=0.41	OR 1.28 (CI: 0.48-3.44) p=0.63
FSFI Satisfaction<5.04	OR 1.22 (CI: 0.38-3.94) p=0.74	OR 2.00 (CI: 0.57-7.06) p=0.28
FSFI Pain<5.51	OR 1.59 (CI: 0.56-4.52) p=0.39	OR 3.50 (CI: 1.04-11.82) p=0.044
FSFI Total<26.55	OR 1.12 (CI: 0.41-3.08) p=0.83	OR 3.28 (CI: 1.06-10.14) p=0.039

## Discussion

In this study, 53.3% of participants had an elevated BMI, 33.8% had an elevated FLI, and 32.5% had elevated HOMA-IR, indicating significant metabolic disorders. Additionally, 92.2% of PCOS patients had increased body hair, and 71.4% showed impaired sexual function, with lower scores across all FSFI domains. Participants with metabolic impairments and BMI ≥25kg/m² reported decreased sexual desire. Multivariate analysis revealed that VAI significantly impacted FSFI-Desire scores (OR 2.56; CI: 1.19-5.56; p=0.016). For FSFI-Satisfaction, higher BMI (OR 3.56; CI: 1.01-12.59; p=0.049) and having a partnership ("no partnership" OR 0.14; CI: 0.03-0.69; p=0.015) were associated with greater satisfaction.

PCOS patients who were in a partnership reported less pain during sexual intercourse compared to single women (OR 0.22; CI: 0.064-0.73; p=0.014), which was also associated with symptoms of depression (FSFI-Pain (OR 3.50, CI: 1.04-11.82; p=0.044)). The symptoms of androgenization such as increased body hair (Ferriman-Gallwey >7 (OR 7.47; CI 1.25-44.74; p=0.028)) played a role in assessing pain during sexual intercourse, as well as in the development of symptoms of anxiety (Ferriman-Gallwey >7 (OR 9.20; CI: 1.02-83.16; p=0.048)). Here too, body image had an influence on sexual experience and even on the development of anxiety.

The results of the HADS questionnaire indicated, on average, a pathological outcome in the anxiety subscale, and elevated scores in the depression scale, although they had not yet reached the pathological range. 41.6% of the study participants had at least mild depression, and 61.0% reported anxiety symptoms. PCOS patients have a higher risk of developing depression or anxiety disorders over the course of their lives - both pre- and postmenopausal [22]. The cause for this may lie in the symptom burden of the patients, but molecular processes of PCOS are also involved [23].

When examining an association between metabolism, sexual experience and mental health, it became apparent that in the univariate analysis two metabolic markers (HOMA-IR>2 (OR 2.57; CI: 1.01-6.54; p=0.047), FLI ≥60 (OR 3.51, CI: 1.19-10.35; p=0.023)) and a low result in the FSFI-Pain-Subscale and in FSFI-Total (FSFI-Pain (OR 3.50, CI: 1.04-11.82; p=0.044), FSFI-Total (OR 3.28; CI: 1.06-10.14; p=0.039)) had an impact on depression in PCOS-patients. In the multivariate analysis, a pathological FLI (FLI≥60 (OR 3.49; CI: 1.14-10.65; p=0.029)) remained associated with depression.

A reduced PSH in patients with PCOS is already known from several studies and is mentioned in the guidelines [3,9]. The PSH of PCOS patients is subject to a myriad of potential influencing factors, given the wide range of symptoms associated with PCOS [9]. Increased androgens and their subsequent androgenization manifestations, such as increased body hair and the presence of a partnership, play an important role in this regard [24]. However, it is self-evident that factors independent of PCOS, such as age, can also influence PSH [25].

Nevertheless, in previous scientific works in the field of PCOS, the influence of metabolism on PSH has not been examined to a comparable depth. While some studies indicate that being overweight and having diabetes can negatively impact PSH, our study also revealed a correlation between these factors [26]. In contrast, the latest meta-analysis by Pastoor et al. found that lean PCOS patients actually exhibited poorer PSH results [9].

The same applies to fertility status [9]. It has previously been shown that infertility can have a negative influence on PSH, as the sexual life of couples undergoing fertility treatment often faces its own challenges and can involve sexual impairments. However, this was not replicated in the meta-analysis by Pastoor et al. [9,27]. Here, fertility status was found to have no significant influence on the PSH of PCOS patients [9].

In addition, women with PCOS exhibited varying results in studies using the FSFI across the individual subdomains of the questionnaire. In most cases, the literature indicates impairment in FSFI-Arousal, FSFI-Orgasm, FSFI-Lubrication, and FSFI-Total, with no impairments in FSFI-Desire [3]. This could not be confirmed in this study, as impairments in all subcategories and FSFI-Total were also observed here.

Although metabolism plays a key role in PCOS patients, there is a lack of studies that thoroughly analyze the connection between PCOS and FLI, VAI, HOMA-IR, and PSH. However, FLI and VAI are often not part of the standard diagnostic procedures in gynecological routine due to additional laboratory parameters such as lipid status. Considering the increased lifetime risk of PCOS patients for conditions such as T2DM, dyslipidemia, or cardiovascular disease, it is important to pay more attention to metabolism, as many chronic diseases result from metabolic changes.

Metabolism also influences the development of depression and anxiety. For example, overweight patients have a higher risk of developing depression [28]. It is already known that women with PCOS have an increased risk of depression and anxiety [3]. This can also be attributed to the increased risk of body image disturbances and low self-esteem associated with PCOS [29,30]. 

Nevertheless, it is important to note that low sexual function was particularly associated with the severity of depression in this cohort. Improving sexual function in women with PCOS could, therefore, potentially reduce the risk of depression. This underscores the value of routinely assessing sexual health using standardized questionnaires, and, where appropriate, referring patients to counselling or psychosexual therapy as part of a multidisciplinary treatment approach.

Strengths and limitations

In this investigation of PCOS and PSH, extensive diagnostic procedures were conducted on the patients to identify potential confounding variables. The diagnostics facilitated the accurate identification of individuals with PCOS. This method ensured that the observed effects were more likely linked to the distinctive characteristics of PCOS and were not impacted by other undisclosed factors.

However, a larger sample size would have been preferable to enhance the statistical certainty of our findings. The participant count could primarily be attributed to difficulties in enrollment. In Germany, women with PCOS who are not seeking to conceive are typically managed by gynecologists in private practices rather than in university clinics, where recruitment for studies is more accessible.

Additionally, the enrollment method employed might have introduced bias to the study cohort. The PCOS patients, who are treated in a university outpatient clinic, may have a more complex or severe medical condition from the outset. Presumably, mainly patients who have been dissatisfied with their previous treatment or who experience more severe impairment of their mental health may respond more or less easily to a call for participation in studies.

Furthermore, incorporating a control group would have provided a more comprehensive understanding of the impact of metabolism on the PSH of women with PCOS. Even if a comparison with the general population is broadly possible due to the validated cut-off values for FLI, BMI, HOMA, HADS, and FSFI, the absence of a control group makes it difficult to determine whether the observed effects are specific to PCOS or reflect broader trends in metabolism and PSH.

Moreover, the potential influence of sociocultural factors and ethnicities on metabolism and PSH may have introduced bias into our study, as these variables could affect both the metabolic markers and the psychosexual outcomes measured, thus complicating the interpretation of our findings and their generalizability across diverse populations. In addition, although weight and metabolism were recorded in our study, body composition, which can have an influence on the individual experience of sexuality, was not.

The present study on PSH could have been influenced by the simultaneous presence of the COVID-19 pandemic during the study period. The pandemic could have caused changes in lifestyle, dietary habits, and social experiences, which in turn potentially affected sexual behavior and mental health. Increased levels of stress and social isolation, known effects of the pandemic, could have heightened mental health challenges such as anxiety and depression, potentially influencing PSH. However, the associations found in our study are likely not influenced by this because the pandemic affected all people simultaneously, thus it could not function as a confounding factor.

## Conclusions

The findings of this study indicate that metabolic dysfunction in women with PCOS - reflected in elevated BMI, FLI, VAI, and HOMA-IR - is closely linked to reduced sexual function and increased symptoms of depression and anxiety. Although this is a cross-sectional study, the findings may still indicate the potential value of incorporating both metabolic and psychosexual assessments into comprehensive PCOS care. Clinicians play a central role in identifying and addressing these interconnected aspects by combining physical evaluations with targeted assessments of sexual and mental health. A more holistic, interdisciplinary approach may improve clinical outcomes and enhance the overall quality of life for patients with PCOS.

## Appendices

**Table 6:** Serological and anthropometric characteristics of the study population Reference range in square brackets, all hormones measured during the follicular phase, cycle days 1-5.

	Mean ± SD
Anti-Müllerian Hormone AMH (ng/ml)	9.2±5.2 [1.2-11.6]
Androstenedione (ng/ml)	3.3±1.6 [0.3-2.2]
C-reactive protein CRP (mg/l)	3.2±4.0 [<5.0]
Creatinine (mg/dl)	0.7±0.09 [0.6-1.0]
Dehydroepiandrosterone sulfate DHEAS (µg/dl)	325.8±155.6 [50.0-511.0]
Estradiol (pg/ml)	43.6±18.7 [30.0-90.0]
Follicle-stimulating hormone FSH (IU/l)	4.7±1.5 [1.4-17.0]
Gamma-glutamyltransferase GGT (U/l)	17.0±7.3 [<38.0]
Glucose 0min (mg/dl)	87.1±7.1
Glucose 60min(mg/dl)	100.4±29.2
Glucose 120min (mg/dl)	89.7±23.8
Height (m)	1.7±0.07
Insulin 0min (mU/l)	11.2±9.9
Insulin 60min (mU/l)	76.5±83.9
Insulin 120min (mU/l)	50.9±59.2
Luteinizing Hormone LH (IU/l)	6.8±5.0 [0.6-89.0]
LH/FSH	1.5±1.1 [<2.0]
Progesterone (ng/ml)	0.3±0.2 [0.1-16.0]
Prolactin (ng/ml)	13.1±6.8 [5.2-26.5]
Sex Hormone-Binding Globulin SHBG (nmol/l)	54.9±33.5 [13.0-71.0]
Testosterone (ng/dl)	51.2±2.5 [12.0-53.0]
